# FGF Signaling Inhibition in ESCs Drives Rapid Genome-wide Demethylation to the Epigenetic Ground State of Pluripotency

**DOI:** 10.1016/j.stem.2013.06.004

**Published:** 2013-09-05

**Authors:** Gabriella Ficz, Timothy A. Hore, Fátima Santos, Heather J. Lee, Wendy Dean, Julia Arand, Felix Krueger, David Oxley, Yu-Lee Paul, Jörn Walter, Simon J. Cook, Simon Andrews, Miguel R. Branco, Wolf Reik

**Affiliations:** 1 Epigenetics Programme, The Babraham Institute, Cambridge, CB22 3AT, UK; 2 Bioinformatics Group, The Babraham Institute, Cambridge, CB22 3AT, UK; 3 Proteomics Research Group, The Babraham Institute, Cambridge, CB22 3AT, UK; 4 Signalling Programme, The Babraham Institute, Cambridge, CB22 3AT, UK; 5 Department of Biological Sciences, Institute of Genetics/Epigenetics, University of Saarland, 66123 Saarbrücken, Germany; 6 Centre for Trophoblast Research, University of Cambridge, Cambridge CB2 3EG, UK; 7 Wellcome Trust Sanger Institute, Hinxton CB10 1SA, UK

## Abstract

Genome-wide erasure of DNA methylation takes place in primordial germ cells (PGCs) and early embryos and is linked with pluripotency. Inhibition of Erk1/2 and Gsk3β signaling in mouse embryonic stem cells (ESCs) by small-molecule inhibitors (called 2i) has recently been shown to induce hypomethylation. We show by whole-genome bisulphite sequencing that 2i induces rapid and genome-wide demethylation on a scale and pattern similar to that in migratory PGCs and early embryos. Major satellites, intracisternal A particles (IAPs), and imprinted genes remain relatively resistant to erasure. Demethylation involves oxidation of 5-methylcytosine (5mC) to 5-hydroxymethylcytosine (5hmC), impaired maintenance of 5mC and 5hmC, and repression of the de novo methyltransferases (Dnmt3a and Dnmt3b) and Dnmt3L. We identify a Prdm14- and Nanog-binding *cis*-acting regulatory region in *Dnmt3b* that is highly responsive to signaling. These insights provide a framework for understanding how signaling pathways regulate reprogramming to an epigenetic ground state of pluripotency.

## Highlights

• Genome-wide analysis of 2i ESCs reveals demethylated genome similar to ICM and PGCs
• Demethylation involves TETs, replicative loss of 5mC and 5hmC, suppression of Dnmt3s
• NANOG/PRDM14 binding element in Dnmt3b enhancer is highly responsive to signaling
• 2i and serum epigenetic signatures exist in populations of NanogGFP ESCs and ICM

## Teaser

The transition to ground state pluripotency involves genome-wide DNA demethylation and oxidation of 5mC to 5hmC by Tet proteins.

## Introduction

**Figure 1:**
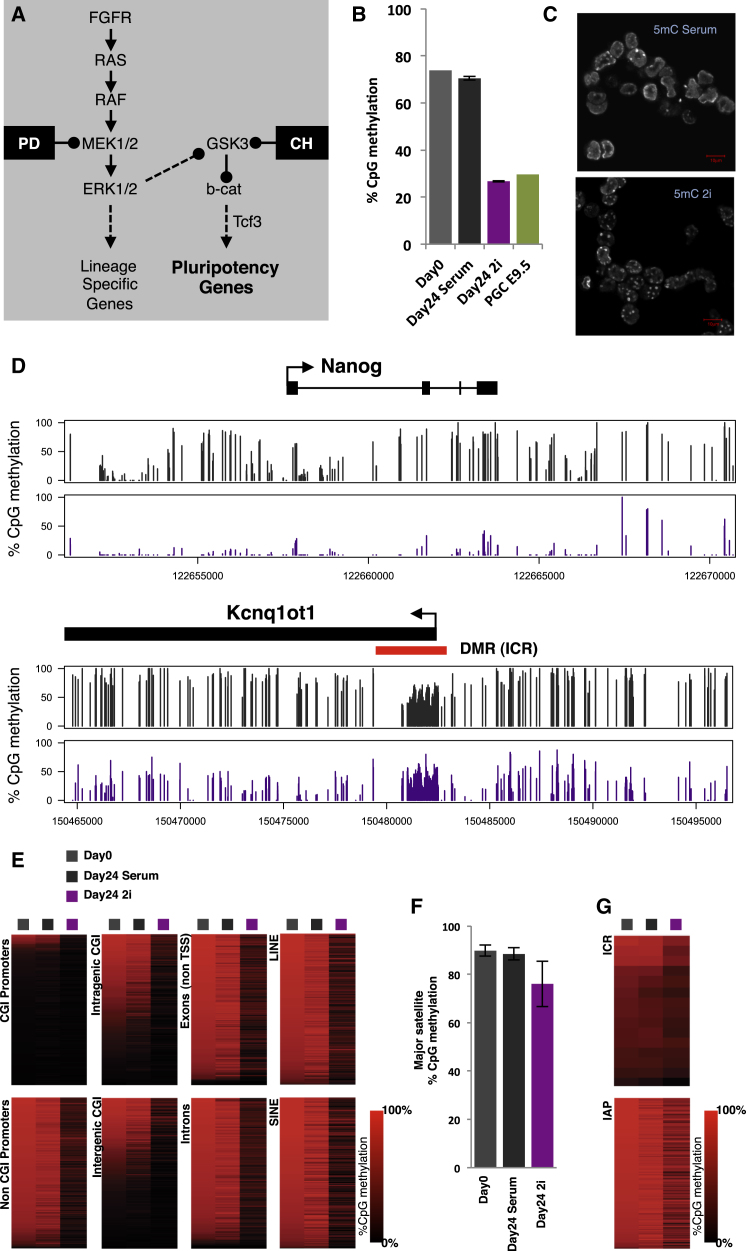
Erk1/2 and Gsk3β Signal Inhibition Induces Global DNA Demethylation (A) Schematic of the signaling pathways inhibited by the “2i” small molecule inhibitors. (B) Global CpG methylation measured by whole-genome BS-seq in serum, 2i, and E9.5 PGCs (data from Seisenberger et al., 2012). Error bars represent the standard deviation in three replicates. (C) Immunofluorescence staining of E14 ESCs with an antibody against 5mC shows reduced euchromatic methylation in 2i while pericentromeric heterochromatic regions maintain high 5mC levels. (D) Example of BS-seq profile in serum (black bars) and 2i (purple bars) ESCs with the *Nanog* locus being strongly demethylated in 2i while the ICR methylation at *Kcnq1ot1* is maintained. (E) Heatmap methylation levels in 500 randomly selected elements (CpG islands (CGIs), Exons, Introns, LINE1, SINE) in Day 0, Day 24 Serum, and Day 24 2i. (F) Confirming IF data, methylation at pericentromeric major satellites remains high in 2i as measured by BS-seq (error bars represent the standard deviation between CpGs). (G) Heatmap methylation levels in 500 randomly selected IAP elements and 15 ICRs. See also Figure S1.

Acquisition of pluripotency in primordial germ cells (PGCs) and the early embryo coincides with genome-wide epigenetic reprogramming of histone modifications and DNA methylation, but the precise relationship between reprogramming and pluripotency is not clear (Seisenberger et al., 2013). Epigenetic reprogramming in PGCs may be induced by signaling pathways such as BMP/Smad (Seisenberger et al., 2013), while FGF signaling in the blastocyst is connected with the exit from pluripotency and epigenetic priming for differentiation (Lanner and Rossant, 2010). It is not well understood how signaling pathways maintain pluripotency in the inner cell mass (ICM), but a distinctive feature of ICM cells is the lack of FGFR2, the earliest functional receptor for FGF4 (reviewed in Lanner and Rossant, 2010). While global erasure of DNA methylation is closely associated with the pluripotent state in PGCs (Seisenberger et al., 2012; Hackett et al., 2013), it appears paradoxical that ICM cells are also globally hypomethylated but ESCs resemble somatic cells in their overall high levels of CpG methylation (Stadler et al., 2011; Smith et al., 2012; Popp et al., 2010). ESCs under standard culture conditions (in fetal calf serum with LIF) receive prodifferentiation signals but are constrained from differentiating by LIF. They have high levels of de novo methyltransferases (Dnmt3a and Dnmt3b), their regulator Dnmt3L, and the hydroxylases Tet1 and Tet2, suggesting continuous reprogramming of their epigenome (Ficz et al., 2011). Because serum cultured ESCs are primed for differentiation by Fgf/Erk, we reasoned that inhibition of this signaling pathway by specific Erk1/2 and Gsk3β inhibitors (2i, Figure 1A) could induce reprogramming to an ICM- or PGC-like epigenetic state. Indeed, work recently published supports this contention by showing that 2i can induce global hypomethylation (as measured by mass spectrometry and at some selected loci in the genome), that the de novo methyltransferases Dnmt3a and Dnmt3b and their regulator Dnmt3L are downregulated in 2i, and that the transcriptional regulator PRDM14 contributes to downregulation of the Dnmt3s and to the maintenance of ESCs in the hypomethylated state (Yamaji et al., 2013; Leitch et al., 2013). The genomic patterns and dynamics, as well as the mechanisms of genome-wide demethylation induced by 2i, remain unknown, and so does the question of whether the extent, patterns, and mechanisms of demethylation occurring in 2i resemble those in preimplantation embryos and PGCs (Seisenberger et al., 2013).

## Results

### Epigenome of Ground State ESCs

To address these questions we carried out genome-wide bisulphite sequencing (BS-seq) and transcriptomics (RNA-seq), comparing ESCs either grown in serum or switched from serum to 2i conditions for 24 days. 2i induced a striking loss of DNA methylation as evaluated by BS-seq (three biological replicates were sequenced for each experimental sample), immunofluorescence, and mass spectrometry (Figures 1B and 1C and Figure 3A); demethylation in 2i was widespread as judged by pairwise individual CpG methylation comparison (Figure S1A available online) and at its maximum resulted in over 95% demethylation. BS-seq showed that this loss of methylation was similar in magnitude to the global methylation erasure that occurs in migratory PGCs prior to their gonadal stage (Figure 1B) (Seisenberger et al., 2012).

**Figure 2:**
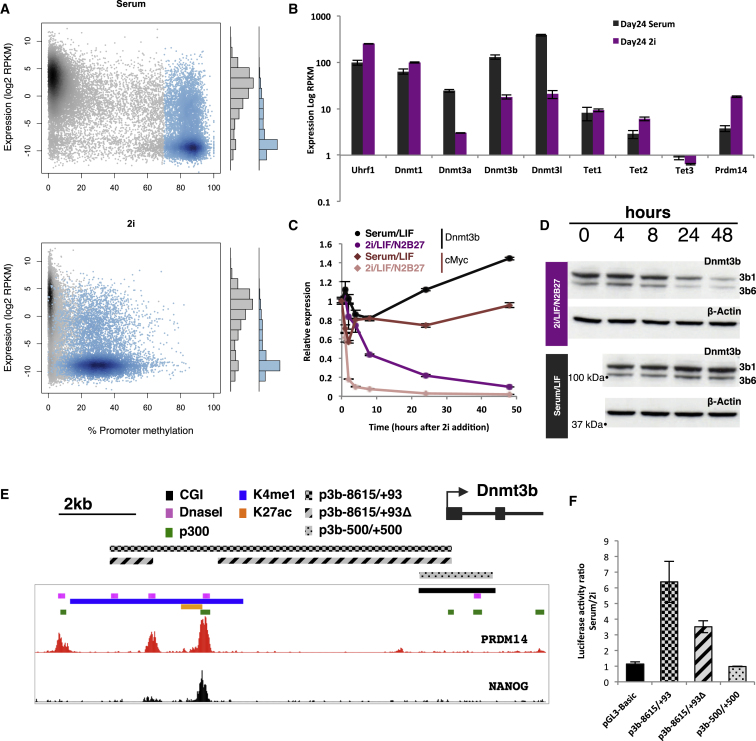
Signaling and Transcriptional Regulation in 2i ESCs (A) Comparison of promoter methylation levels with corresponding gene expression shows that in serum ESCs highly methylated genes are mostly underexpressed (blue group of gene promoters, blue histogram). In 2i (lower panel), there is no global change in the distribution of gene expression (see histograms on the right hand side). (B) Substantial downregulation of Dnmt3a, Dnmt3b, and Dnmt3L in ESCs cultured in 2i for 24 days as measured by RNA-seq (error bars represent the range of values in two biological replicates). Transcription of maintenance methylation components Dnmt1 and Uhrf1 is not reduced, while Tet2 and Prdm14 expression are upregulated in 2i ESCs. (C) Timecourse of downregulation of Dnmt3b in 2i versus serum ESCs. Note the rapid downregulation of cMyc, a direct target of the Erk1/2 signaling pathway (error bars represent the range of values in two biological replicates). (D) DNMT3B protein levels at different time points after 2i addition with visible decrease at 24 hr after 2i. (E) *Dnmt3b* upstream promoter genomic region shows PRDM14 and NANOG binding peaks overlapping putative enhancer elements. Shaded horizontal bars indicate the constructs used in the luciferase assay in (F). (F) Luciferase reporter assay with no promoter (pGL3-Basic) or the *Dnmt3b* promoter p3b −8615/+93 showing strong inhibition of luciferase expression in 2i. Deletion of Prdm14 and Nanog binding sites (p3b −8615/+93Δ) reduces repression in 2i by 50% (error bars represent the standard deviation of four independent transfections for each plasmid in each condition, except the p3b−500/+500 with two independent transfections).

**Figure 3:**
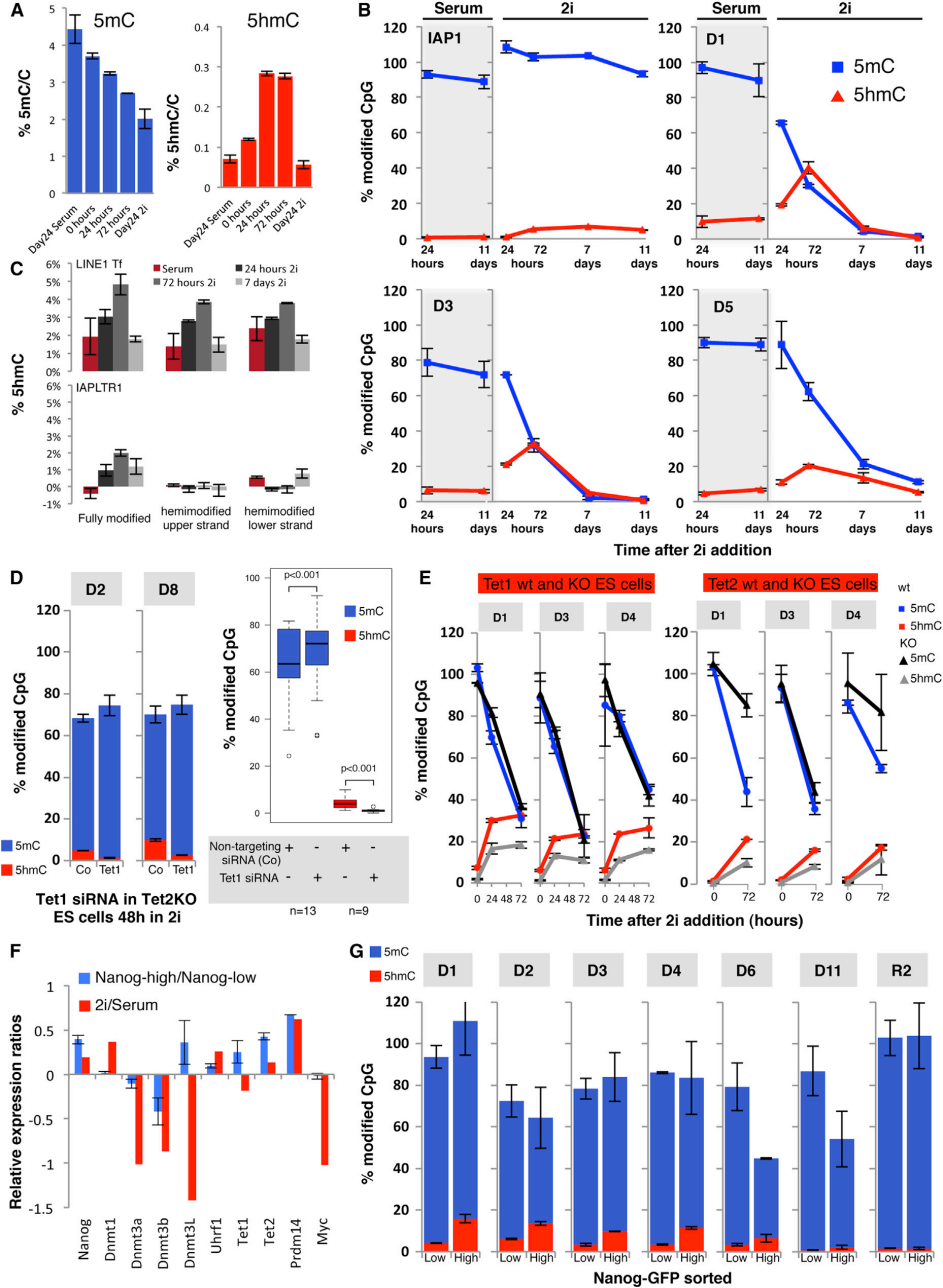
Demethylation in 2i Involves Hydroxylation (A) Mass spectrometric measurement of global 5mC and 5hmC levels in E14 (Day 24 Serum and 2i) and *Tet1wt* ESCs at 0, 24, and 72 hr after 2i addition showing a stepwise decline of 5mC and a substantial transient increase in 5hmC. (B) Absolute 5mC (blue) and 5hmC (red) levels in individual CpGs measured by GlucMS-qPCR validate the BS-seq data and show that demethylating CpGs are significantly hydroxymethylated after 2i addition with a peak at 72 hr. CpGs were selected according to the extent of demethylation: not demethylated (IAP1) and demethylated in 2i (D1, D3, and D5). (C) Hairpin bisulphite and oxidative bisulphite sequencing in LINE1Tf 5′UTR at 24 hr, 72 hr, and 7 days after 2i addition showing the extent of hemimethylation through early demethylation phase with a 5hmC peak at 72 hr in LINE1Tf, but not IAP long terminal repeat 1 (IAPLTR1). (D) GlucMS-qPCR in *Tet2* KO with Tet1 knockdown ESCs shows increased 5mC levels 48 hr after 2i addition (boxplot, blue bars, paired t test on 13 demethylating targets) and virtually no 5hmC acquisition compared to the control (boxplot, red bars). Individual examples are shown to the left. (E) GlucMS-qPCR in *Tet1wt* versus *Tet1*^*−/−*^ and *Tet2wt* versus *Tet2*^*−/−*^ shows demethylation in some CpG sites but significantly delayed demethylation in others. (F) Gene expression measurement in NanogGFP sorted ESCs and similarities with 2i versus serum. (G) GlucMS-qPCR in NanogGFP sorted ESCs. Error bars represent the range of values in two biological replicates throughout. See also Figures S2–S4.

Substantial demethylation occurred in most genomic contexts, including gene bodies (Figures 1D and 1E), non-CpG island (non-CGI) promoters that are substantially methylated in serum ESCs, and the SINE and LINE1 transposon families (Figure 1E). While there was some erosion of methylation in major satellites, intracisternal A particles (IAPs), and imprinting control regions (ICRs), they remained ultimately resistant to erasure (Figures 1D, 1F, and 1G, Figure 3B, Figure S2). This pattern of demethylation closely resembles that of reprogramming in the preimplantation embryo, with demethylation of LINE1 retrotransposons and single copy genes such as *Nanog*, maintenance of IAP and ICR methylation (Tomizawa et al., 2011), and detectable pericentromeric heterochromatin methylation (methylation in major satellites) in ICM cells (Santos et al., 2002). Notably, this pattern of demethylation is also characteristic of PGCs during their migration phase (Seisenberger et al., 2012), while ICRs and germline-specific genes are demethylated upon their arrival in the gonads (Seisenberger et al., 2012; Guibert et al., 2012; Hajkova et al., 2002). As in PGCs, global demethylation in 2i did not result in promiscuous transcription of demethylated genes (Figure 2A). Indeed, a substantial number of genes in serum cultured ESCs have promoters that are highly methylated with the majority of them being transcriptionally silenced (blue group of promoters in Figure 2A); while these promoters are demethylated in 2i, this does not result in upregulation of the associated genes.

### Rapid Dnmt3b Transcriptional Changes Are Effected through a 2i-Responsive *cis*-Element

Examining the transcriptome we confirmed (Marks et al., 2012; Yamaji et al., 2013; Leitch et al., 2013) substantial downregulation of the two de novo methyltransferases Dnmt3a and Dnmt3b and their regulator Dnmt3L in 2i (Figure 2B); in contrast, expression levels of the maintenance methyltransferase Dnmt1 and its targeting factor Uhrf1 were not reduced (Figure 2B). DNA hydroxylase Tet2 levels were elevated consistently in 2i while Tet1 and the lowly expressed Tet3 showed some variability between different ESC lines (data not shown), but on the whole they were not significantly altered (Figure 2B). These transcriptional changes (together with changes in protein levels) occurred within the first 24 hr of 2i addition (Figures 2C and 2D and data not shown); considering this rapid decline we asked if downregulated genes were direct targets of the Erk1/2 and Gsk3β pathways, similar to cMyc (a prototypical early response gene of the Erk1/2 pathway). Dnmt3b plays a major role in de novo methylation in the early embryo (Okano et al., 1999), therefore we chose to focus on its regulation in 2i. Dynamic downregulation of Dnmt3b mRNA occurred within 4–8 hr of 2i addition (but not as fast as cMyc, which declines rapidly in 2i due to loss of Erk signals driving cMyc transcription and rapid decay of preexisting cMyc mRNA; Figure 2C). DNMT3B protein levels declined substantially 24 hr after 2i addition (Figure 2D) and were barely detectable at later stages (the same was true of DNMT3A, data not shown). Since transcriptional downregulation of Dnmt3b was somewhat slower than that of cMyc, we considered the possibility that Dnmt3b silencing was carried out by a *cis*-acting transcriptional regulator, and we therefore transfected a luciferase expression construct driven by the *Dnmt3b* promoter (including 8 kb of upstream sequence; Figure 2E) into serum or 2i cultured ESCs. We found that 2i caused a 5-fold reduction in expression of luciferase compared to ESCs in serum (Figure 2F), indicating that most of the transcriptional difference seen in vivo (Marks et al., 2012; Yamaji et al., 2013; Leitch et al., 2013) reflects signaling input into a *cis*-acting element. No difference in transcription was seen between serum and 2i when a ±0.5 kb (around the transciptional start site) *Dnmt3b* promoter region was driving luciferase activity (Figure 2F). Candidates for regulatory sequences within the 8 kb transfected fragment include a prominent cluster of enhancer hallmarks (DNaseI, H3K4me1, H3K27ac, and p300) and binding of transcription factors Nanog and Prdm14 upstream of the *Dnmt3b* promoter, with Prdm14 being highly upregulated in 2i cultured ESCs (Figures 2B and 2E). Notably, deletion of the Prdm14/Nanog binding region reduced the difference in the response to serum versus 2i of the *Dnmt3b* construct by 50% (Figure 2F). This is consistent with significant upregulation of Dnmt3b in *Prdm14* knockout or knockdown ESCs (Yamaji et al., 2013; Ma et al., 2011), and furthermore with increased methylation in *Prdm14* knockout ESCs cultured in 2i (Yamaji et al., 2013; Leitch et al., 2013).

### 2i Demethylation Involves Oxidation, Replicative Loss, and Repression of De Novo Methyltransferases

Given the rapid downregulation of Dnmt3b, Dnmt3a, and Dnmt3L and increased expression of Tet2, we examined the precise dynamics of demethylation and hydroxymethylation from the beginning of the 2i treatment. At the global level, methylation decreased substantially in a stepwise manner during the first 24 and 72 hr after 2i addition, while during the same period there was a more than 2-fold increase in hydroxymethylation (Figure 3A). This global increase in hydroxymethylation was transient and declined in consecutive passages in parallel to the loss of methylation. We next examined five individual genomic regions that underwent substantial demethylation (>80%) and five control regions that were resistant to demethylation (including two IAPs and two ICRs), analyzing in each case the levels of 5mC and 5hmC at one MspI site (CCGG) at 24 hr, 72 hr, 7 days, and 11 days after 2i addition by glucosylation of genomic 5hmC followed by methylation-sensitive qPCR (GlucMS-qPCR) (Figure 3B, Figure S2). In all demethylating regions there was a steep loss of 5mC that occurred linearly during the early part of this time period, with demethylation more or less completed 7 days after 2i. Resistant regions experienced small losses of methylation during this period but stabilized during prolonged culture in 2i. Notably, all regions that demethylated acquired substantial levels of 5hmC (up to 40% resistance of the glucosylated DNA to MspI digestion) with a peak at 72 hr after 2i, which then declined together with 5mC, while regions that did not demethylate acquired only minimal amounts of 5hmC (Figure 3B, Figure S2). While expression levels of Dnmt1 (confirming Marks et al., 2012 and Leitch et al., 2013) and Uhrf1 were not reduced during 2i induction, we wished to determine if maintenance of 5mC or 5hmC was nevertheless impaired in demethylating regions, and thus carried out hairpin bisulphite and oxidative bisulphite sequencing on LINE1 elements and on IAPs (Figure 3C, Figure S3). Hairpin bisulphite sequencing allows simultaneous examination of CpG methylation on both DNA strands (identifying both fully and hemimethylated CpG dyads) while oxidative bisulphite sequencing discriminates between 5mC and 5hmC (Arand et al., 2012) within the same CpG dyad. A maximum amount of 5hmC was acquired by 72 hr after 2i at hemimodified CpG dyads of LINE1Tf 5′UTR (which demethylate) but not at IAPs (which do not), consistent with reduced maintenance of both 5mC and 5hmC in LINE1Tf (Figure 3C). A small amount of 5hmC accumulated on fully modified CpGs in IAPs (also detected by GlucMS-qPCR, Figure 3B) consistent with the mild erosion of IAP methylation in 2i and potentially indicating a mechanism whereby some of the 5hmC can be selectively “read” at replication as 5mC, a process that might be locus specific. Similar observations have been reported on ICRs and on loci escaping reprogramming in PGCs (Piccolo et al., 2013; Hackett et al., 2013).

The regulated hydroxylation during demethylation implicates the Tet hydroxylases in this process; indeed, regions in the genome that were most demethylated were enriched for Tet1 binding sites (Figure S1B). In order to eliminate most 5hmC in ESCs, we knocked down Tet1 with siRNA in *Tet2* knockout ESCs, followed this with 2i treatment for 48 hr, and measured 5mC and 5hmC levels by GlucMS-qPCR. Loss of Tet1 and Tet2 resulted in substantial reduction of 5hmC acquisition in all demethylating regions, and it delayed demethylation in 8 out of 13 regions tested (Figure 3D). Preliminary results with individual *Tet1* and *Tet2* knockout ESCs subjected to 2i treatment suggest that while hydroxylation is impaired in all demethylating regions, demethylation is impaired in some, but not in others (Figure 3E). In order to expand this preliminary finding, more detailed genome-wide analyses of methylation and hydroxymethylation at different time points after 2i induction and upon manipulation of Tet1 and Tet2 levels will need to be carried out. We considered the possibility that the rapid decline of the de novo methyltransferases allowed the Tet enzymes increased access to the 5mC substrate; however, combined knockdown of Dnmt3a, Dnmt3b, and Dnmt3L in serum/LIF cultured ESCs did not result in increased hydroxylation. While knockdown of Dnmt3 proteins led to demethylation of LINE1Tf sequences, it had only mild effects on single copy loci that demethylate in 2i (Figure S4), indicating that downregulation of the de novo methyltransferases does not mimic the methylation loss seen in the serum to 2i transition at all genomic loci. Indeed, it has been previously observed that *Dnmt3a/Dnmt3b* double knockout ESCs lose global methylation slowly over consecutive passages (Jackson et al., 2004; Arand et al., 2012), while Dnmt3a and Dnmt3b are needed to maintain high levels of methylation at LINE1s due to inefficient methylation maintenance at these regions (Arand et al., 2012). It is possible that 2i treatment causes increased accessibility of chromatin to Tet enzymes (Nakamura et al., 2012) and hence the substantial oxidation of DNA.

### Heterogeneity in Serum ESCs Involves a Fluctuating Epigenome

ESCs grown in serum show heterogeneous and dynamic expression of Nanog, Rex1, Stella, and other pluripotency-related genes, while 2i ESCs express these genes more homogenously (Chambers et al., 2007; Wray et al., 2010). A previous study compared Rex1GFP-high and -low subpopulations from serum ESCs, and found that, on a transcriptional level, the Rex1GFP-high cells were more similar to ESCs grown in 2i (Marks et al., 2012). We used fluorescence-activated cell sorting (FACS) to isolate NanogGFP-high and -low subpopulations from a NanogGFP (targeted Nanog-GFP clone A, or TNGA) ESC line. We found that NanogGFP-high ESCs had elevated expression of Prdm14 and Tet2 and reduced expression of Dnmt3b, in comparison to NanogGFP-low cells (Figure 3F). We examined several loci that demethylate in 2i, and found that all of these had increased hydroxylation and one-third had reduced methylation in NanogGFP-high cells relative to NanogGFP-low cells (Figure 3G). This suggests that epigenetic fluctuations including methylation and demethylation brought about by altered signaling contribute to the metastable cell states in populations of serum ESCs. While NanogGFP-high ESCs display transcriptional and epigenetic similarities to ESCs grown in 2i, not surprisingly, these two cell states are not exactly equivalent. Dnmt3L expression is reduced in 2i but elevated in NanogGFP-high ESCs (Figure 3F), and the magnitude of methylation changes in 2i is greater than in the NanogGFP-high subpopulation (Figure 3G).

### 2i Methylome Resembles that of ICM and Migratory PGCs

**Figure 4:**
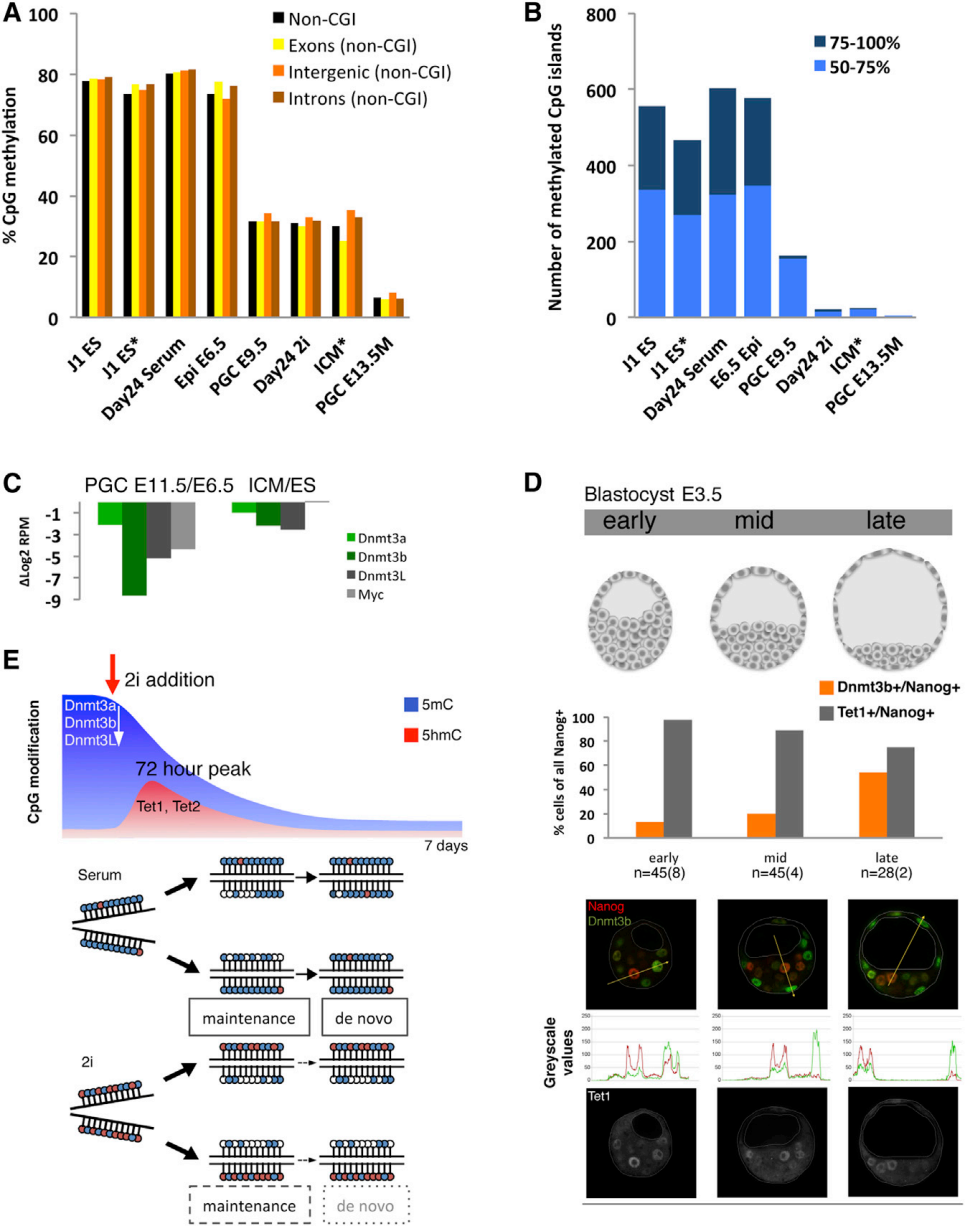
Global Hypomethylation in 2i ESCs Resembles that of ICM Cells and Migratory PGCs (A) Global methylation levels in ESCs, ICM cells, and PGCs from whole-genome BS-seq data and reduced representation BS-seq (RRBS) data sets (RRBS data are labeled with asterisk) (Seisenberger et al., 2012; Smith et al., 2012). (B) Number of highly methylated (75%–100%) or moderately methylated (50%–75%) CGIs of the same samples in (A). (C) Expression of de novo methyltransferases Dnmt3a, Dnmt3b, Dnmt3L, and Myc in PGC E11.5 compared to E6.5 epiblast cells and ICM versus ESCs (Tang et al., 2010; Seisenberger et al., 2012). (D) E3.5 blastocysts stained for NANOG (red), DNMT3B (green), and TET1 (white). Mouse E3.5 blastocysts were collected from CD1 × CD1 and (C57BL/6 × CBA/Ca) × 129/Sv crosses and were classified as early, mid, or late E3.5, depending on their size and extent of cavitation. Late blastocysts show higher DNMT3B expression as well as a greater proportion of ICM cells expressing both NANOG and DNMT3B and slightly fewer NANOG- and TET1-expressing cells (the number of Nanog-positive cells counted from the total number of embryos in parenthesis is shown below the barplot). RGB profiles for representative ICM (NANOG high) and outer trophectoderm (TE) cells are shown for each stage. (E) Model for 2i-mediated demethylation including a peak of oxidation and reduced efficiency of maintenance methylation and disruption of de novo methylation.

We next asked to what extent the epigenome in 2i ESCs reflected that of early embryos. To do this, we compared our methylome data sets to BS-seq from PGCs (Seisenberger et al., 2012) and reduced representation bisulphite sequencing (RRBS-seq) (Smith et al., 2012) from the early embryo (Figures 4A and 4B). Because CGIs are overrepresented in RRBS-seq data, we divided all data sets into a non-CGI and a CGI component, which makes the two methods more comparable. For example, the non-CGI component of RRBS-seq compared to BS-seq of serum-grown ESCs shows very similar methylation levels in various genomic elements (Figure 4A). Notably, methylation levels of 2i ESCs at these same regions are highly similar to those of ICM cells and E9.5 migratory PGCs, and hence distinct from serum ESCs and epiblast on the one hand, and from E13.5 gonadal PGCs on the other. The ICM, PGC, and 2i ESC methylomes also closely resembled each other at the level of CGIs: both showed greatly reduced numbers of methylated CGIs compared to serum cultured ESCs and epiblast cells (Figure 4B), further supporting the idea that epigenetically, 2i ESCs are a model for preimplantation embryos (especially ICM) and for migratory PGCs. A more detailed examination of the methylation patterns in these CGIs revealed that while the majority are demethylated, those CGIs that retained greater than 25% methylation in 2i ESCs, PGCs, and ICM corresponded to highly methylated CGIs from their cell type of origin (serum ESCs, epiblast, and oocytes, respectively), a result illustrated by hierarchical clustering of the data sets (Figure S1C). This implies that any remaining qualitative differences between the erased methylomes of 2i ESCs, PGCs, and ICM are related to their developmental history as opposed to differences in the mechanism by which they became demethylated. We were unable to find any compelling transcriptomic similarities between the three globally demethylated cell types (data not shown). Global uncoupling of methylation and gene expression is characteristic of PGCs (Seisenberger et al., 2012) and 2i ESCs (Figure 2A) and apparently also true of the ICM. This uncoupling thus seems characteristic of the epigenetic ground state of pluripotency.

The globally hypomethylated state in 2i ESCs, PGCs, and ICM is characterized by suppression of Dnmt3a, Dnmt3b, and Dnmt3L (Figure 4C and Yamaji et al., 2013; Leitch et al., 2013). Therefore in their simplest form the 2i-to-serum epigenetic transition is characterized by increased expression of Dnmt3b in cells that express Nanog and Tet1. Can such a pattern be found in the ICM to epiblast transition in vivo? In E3.5 ICM cells Tet1 and Nanog expression is indeed high while Dnmt3b is low, whereas in the epiblast Dnmt3b is highly expressed while Tet1 and Nanog have been silenced (Tang et al., 2010). We therefore examined E3.5 early, mid, and late blastocysts (depending on their size and extent of cavitation) for NANOG, DNMT3B, and TET1 expression by immunofluorescence (Figure 4D). Early and mid E3.5 ICM cells that prominently express NANOG and TET1 rarely express DNMT3B. A notable transition occurs in late-stage ICM cells that now express DNMT3B prominently together with moderately high TET1 and NANOG. Interestingly, some NANOG-positive late ICM cells have lost high expression of TET1, potentially indicating an early epigenetic change in the transition to epiblast. This suggests that early ICM cells resemble 2i ESCs in their ground state epigenome, while later stage ICM cells resemble serum ESCs before transiting to an epigenetically primed epiblast cell type that is characterized by high levels of Dnmt3b with Tet1 (and Tet2) being silenced (Tang et al., 2010).

## Discussion

Work just published has shown that 2i can induce global hypomethylation in ESCs and that Dnmt3a, Dnmt3b, and Dnmt3L and their regulation by Prdm14 is involved in this demethylation (Yamaji et al., 2013; Leitch et al., 2013). We have expanded on these findings by carrying out genome-wide BS-seq and RNA-seq studies and by examining the mechanisms of demethylation and the resemblance of the reprogrammed state to PGCs, ICM, and the subpopulation of serum ESCs that express high levels of Nanog. The key conclusions from this work are that demethylation occurs rapidly upon 2i induction and in extent and pattern remarkably resembles the epigenomes of migrating PGCs and of ICM cells in the blastocyst, with imprinted genes being particularly resistant to erasure in all three systems. Hence demethylation in imprinted ICRs can apparently be uncoupled from genome-wide erasure of methylation.

Mechanistically, demethylation involves at least three components, with Tet1 and Tet2 converting 5mC to 5hmC, together with replicative loss of 5mC and 5hmC (Inoue and Zhang, 2011) or its further processing (Ito et al., 2011; He et al., 2011), while at the same time de novo methylation is disabled (Figure 4E). These mechanistic components and associated characteristic gene expression changes (Figure 2B and Figure 4C) closely resemble those implicated in demethylation in migratory PGCs (including cMyc, a signature response in 2i) and in preimplantation embryos (Seisenberger et al., 2012; Hackett et al., 2013; Kagiwada et al., 2013; Piccolo et al., 2013; Grabole et al., 2013). The 2i system therefore provides a convenient model in which to study detailed interactions between these mechanisms. As a demonstration of this, using luciferase assays in 2i ESCs, we identified a *cis*-acting element that connects changes in Erk1/2 and Gsk3β signaling with Dnmt3b expression (Figures 2E and 2F), a mechanism likely to also be functional in the ICM and PGCs.

In the ICM and in serum cultured ESCs, the two pluripotent cell populations are short lived with rapid transitions between them being intricately regulated by Erk1/2 and Gsk3β signaling and the epigenetic networks described here, as well as the linked pluripotency transcription factor network. Our findings establish a signaling principle for the epigenetic ground state of pluripotency in which Erk1/2 and Gsk3β signaling dynamically regulates the DNA methylation machinery, with the combined effects of downregulation of the de novo system and widespread DNA hydroxylation destabilizing the maintenance system, leading to inefficient propagation of DNA methylation and ultimately global demethylation (Figure 4E).

## Experimental Procedures

### Cell Lines, Culturing Conditions, and Blastocysts

E14, *Tet1*^*−/−*^, *Tet1wt*, *Tet2*^*−/−*^, and *Tet2wt* ESCs were cultured without feeders either in standard serum-containing media (DMEM 4,500 mg/l glucose, 4 mM L-glutamine, 110 mg/l sodium pyruvate, 15% fetal bovine serum, 1 U/ml penicillin, 1 μg/ml streptomycin, 0.1 mM nonessential amino acids, 50 μM β-mercaptoethanol, and 10^3^ U/ml LIF ESGRO) or under 2i culturing conditions (Ying et al., 2008) (serum-free N2B27 [Cat. DMEM/F12: GIBCO 21331; Neurobasal: GIBCO 21103; N2: Stem Cells SF-NS-01-005; B27: GIBCO 17504-044] supplemented with 10^3^ U/ml LIF and Mek inhibitor PD0325901 [1 μM] and Gsk3β inhibitor CHIR99021 [3 μM]). NanogGFP (TNGA) ESCs (Chambers et al., 2007) were cultured with feeders in standard serum-containing media. FACS was performed after cells were stained with an antifeeder antibody (Miltenyi Biotech) and DAPI on a BD FACSAria or BD Influx instrument. Mouse E3.5 blastocysts were collected from CD1 × CD1 and (C57BL/6 × CBA/Ca) × 129/Sv crosses.

### DNA/RNA Extraction and RNA-seq and BS-seq Library Preparation

Genomic DNA was prepared using QIAamp DNA Micro Kit or AllPrep DNA/RNA mini kit (QIAGEN). RNA was extracted using either the AllPrep DNA/RNA mini kit or RNeasy mini kit (QIAGEN) and subjected to DNAase treatment using the Ambion DNA-free kit according to the manufacturers’ instructions. mRNA library preparation for RNA-seq was done as previously described (Ficz et al., 2011). For BS-seq library preparation DNA samples were fragmented by sonication (Covaris) and adaptor ligated (using Illumina supplied methylated adaptors and NEBnext library preparation kit). Subsequently, DNA was bisulphite-treated using the Sigma Imprint kit, according to the manufacturer’s instructions (one step protocol). Final library amplification (16 cycles) was done using Pfu Turbo Cx (Agilent), after which the libraries were gel-purified using QIAGEN Minelute kit.

### GlucMS-qPCR Assay

Preparation of gDNA for GlucMS-qPCR assay was done as described previously with modifications (Ficz et al., 2011). Briefly, 250 ng of genomic DNA was treated with T4 Phage β-glucosyltransferase (NEB M0357S) according to the manufacturer’s instructions. Seventy nanograms of glucosylated gDNA were digested with 10 U of HpaII or MspI or no enzyme (mock digestion) at 37°C for 2 hr, then inactivated for 20 min at 80°C. The HpaII- and MspI-resistant fraction was quantified by qPCR using primers designed around a single HpaII/MspI site, normalizing to the mock digestion control (Table S2).

### DNA Sequencing

Libraries were sequenced on either an Illumina GAIIx or an Illumina HiSeq using the default RTA analysis software. See Table S1 for the outcomes of the sequencing runs.

## Accession Numbers

Raw data from all libraries was deposited with GEO under the accession GSE42923.

## Supplemental Information

Document S1. Supplemental Experimental Procedures, Figures S1–S4, and Tables S1 and S2
