# Supplementary material for: FGF Signaling Inhibition in ESCs Drives Rapid Genome-wide Demethylation to the Epigenetic Ground State of Pluripotency
Source: Cell Stem Cell. 2013 Sep 5;13(3):351–9. doi: 10.1016/j.stem.2013.06.004 (PMC3765959; doi:10.1016/j.stem.2013.06.004)
Supplement: Document S1. Supplemental Experimental Procedures, Figures S1–S4, and Tables S1 and S2 [file mmc1.pdf]

Cell Stem Cell, volume *13*

Supplemental Information

FGF Signaling Inhibition in ESCs Drives Rapid Genome-wide Demethylation to the Epigenetic Ground State of Pluripotency

Gabriella Ficz, Timothy A. Hore, Fatima Santos, Heather J. Lee, Wendy Dean, Julia Arand, Felix Krueger, David Oxley, Yu-Lee Paul, Jörn Walter, Simon J. Cook, Simon Andrews, Miguel R. Branco, and Wolf Reik

Supplementary information Ficz et al. 2013

Inventory of supplementary information:

The supplementary information contains four figures with legends, two tables, additional experimental methods and method references. Figure S1 relates to Figure 1 and contains additional genome-wide methylation analysis for the Serum-2i comparison, hierarchical clustering of methylation data in ES cells and other embryonic developmental stages and correlation of methylation with Tet1 binding sites in ES cells. Figures S2-4 extend Figure 3 and include additional details related to the mechanism of demethylation in 2i. Table S1 details the Illumina sequencing run statistics and Table S2 lists the primers and sequences used in this work.

Supplementary Figures

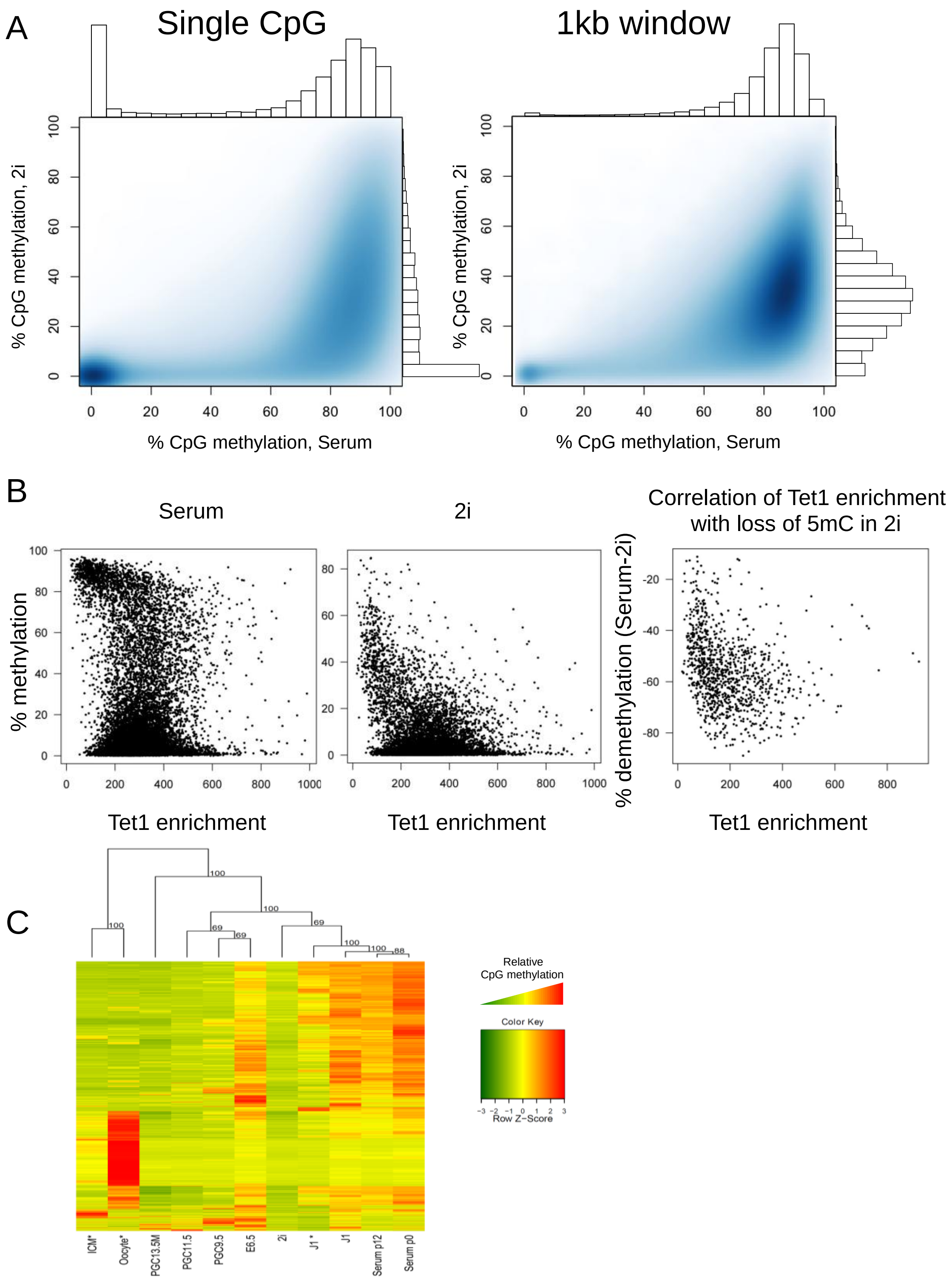

Figure S1. Genome-wide analysis of Serum and 2i ES cells (related to Figure 1). **A.** Pairwise comparison of CpG methylation in Serum and 2i. Density histograms are displayed in both samples. **B.** Correlation of Tet1 ChIP enrichment with CpG island methylation levels in Serum and 2i. Tet1 enrichment (horizontal axes) is compared to the corresponding CGI methylation levels in Serum and 2i (vertical axes). Strongly demethylated CGIs show increased Tet1 enrichment (lower graph). **C.** Hierarchical clustering of CGI methylation in ESCs and embryos. Each of the demethylated cell types (2i ESCs, PGCs and ICM) group preferentially with their respective cell type of origin (serum ESC, epiblast and oocyte) following hierarchical clustering of normalised CGI methylation values (see tree above). Bootstrap assessment of each cluster is indicated and was performed with 10,000 replicates.

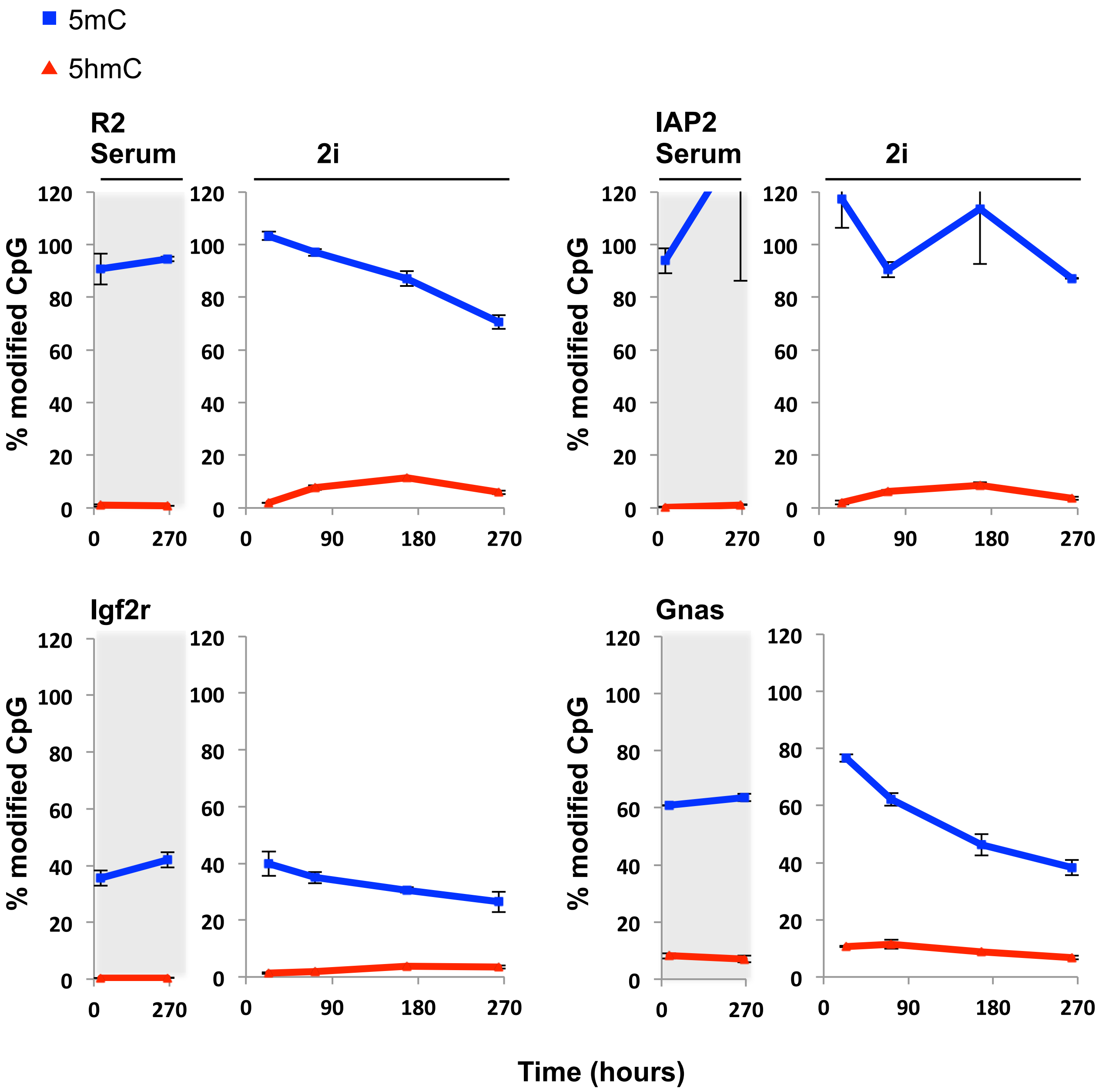

5mC
5hmC
R2
Serum
2i
IAP2
Serum
2i
Igf2r
Gnas
% modified CpG
0
90
180
270
Time (hours)

Figure S2. Genomic targets resisting demethylation in 2i (related to Figure 3). CpG methylation assessed by glucosylation based methylation sensitive qPCR (Gluc MS-qPCR). 5mC (blue) and 5hmC (red) levels in Serum are shown in shaded panels and next to each are the corresponding values in the 2i at different times after 2i addition. Error bars represent the range of values in two biological replicates throughout.

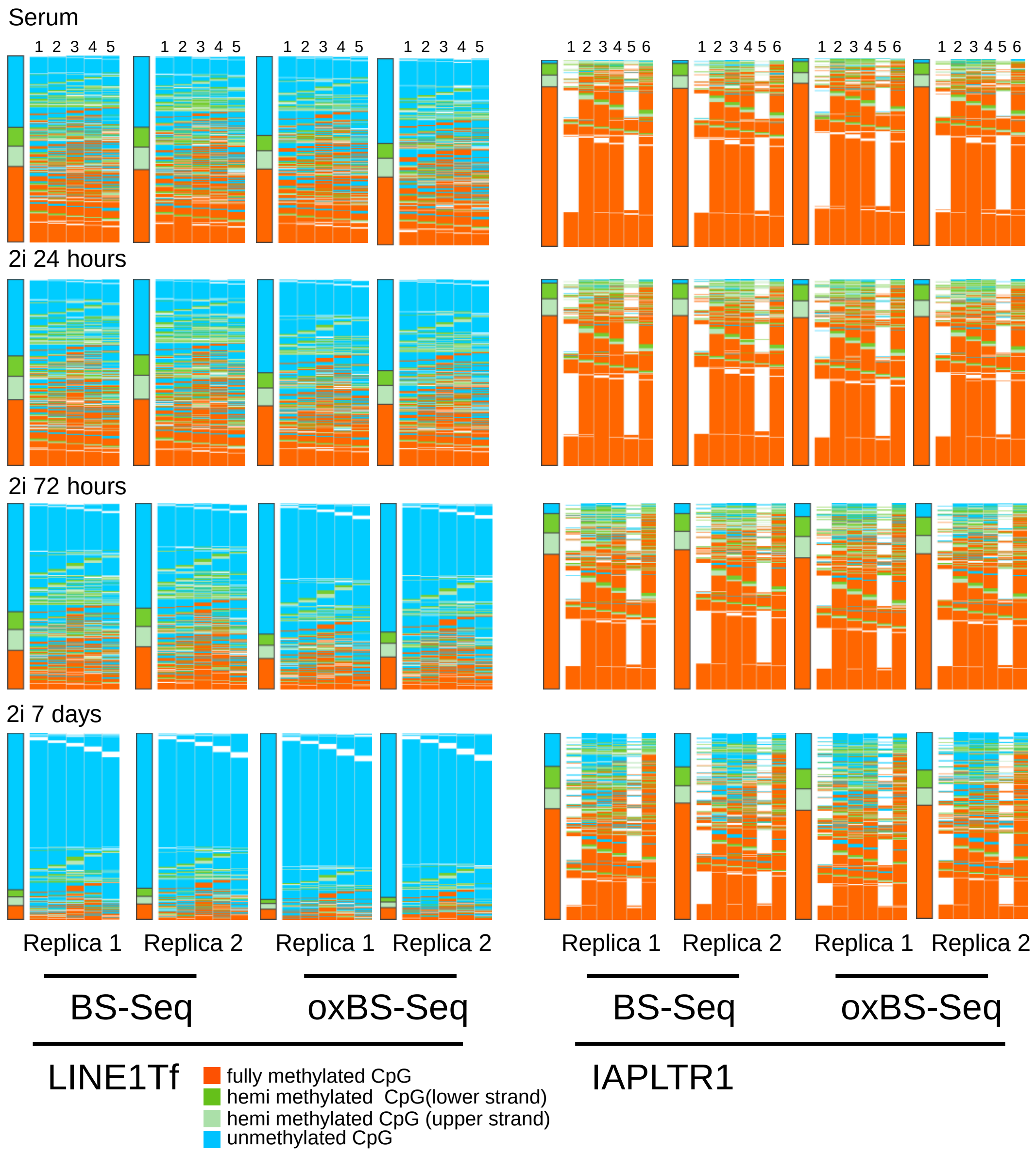

Serum
2i 24 hours
2i 72 hours
2i 7 days
1 2 3 4 5
1 2 3 4 5 6
Replica 1
Replica 2
BS-Seq
oxBS-Seq
LINE1Tf
IAPLTR1
fully methylated CpG
hemi methylated CpG(lower strand)
hemi methylated CpG (upper strand)
unmethylated CpG

Figure S3. Oxidative hairpin bisulfite sequencing of LINE1Tf 5'UTR and IAPs (related to Figure 3). Individual CpGs (numbered 1-5 or 1-6 on the top of the figure) of the consensus sequence are displayed according to the type of methylation in the CpG dyad: orange is fully methylated, greens are hemimethylated and blue is unmethylated. The oxBS-Seq represents absolute 5mC levels while the BS-Seq is measuring both 5mC+5hmC. Concentrations of all species within the whole genome are shown on the left side of each panel for Serum cultured E14 ES cells (top panels) and below at different timepoints in 2i (24, 72 hours and 7days).

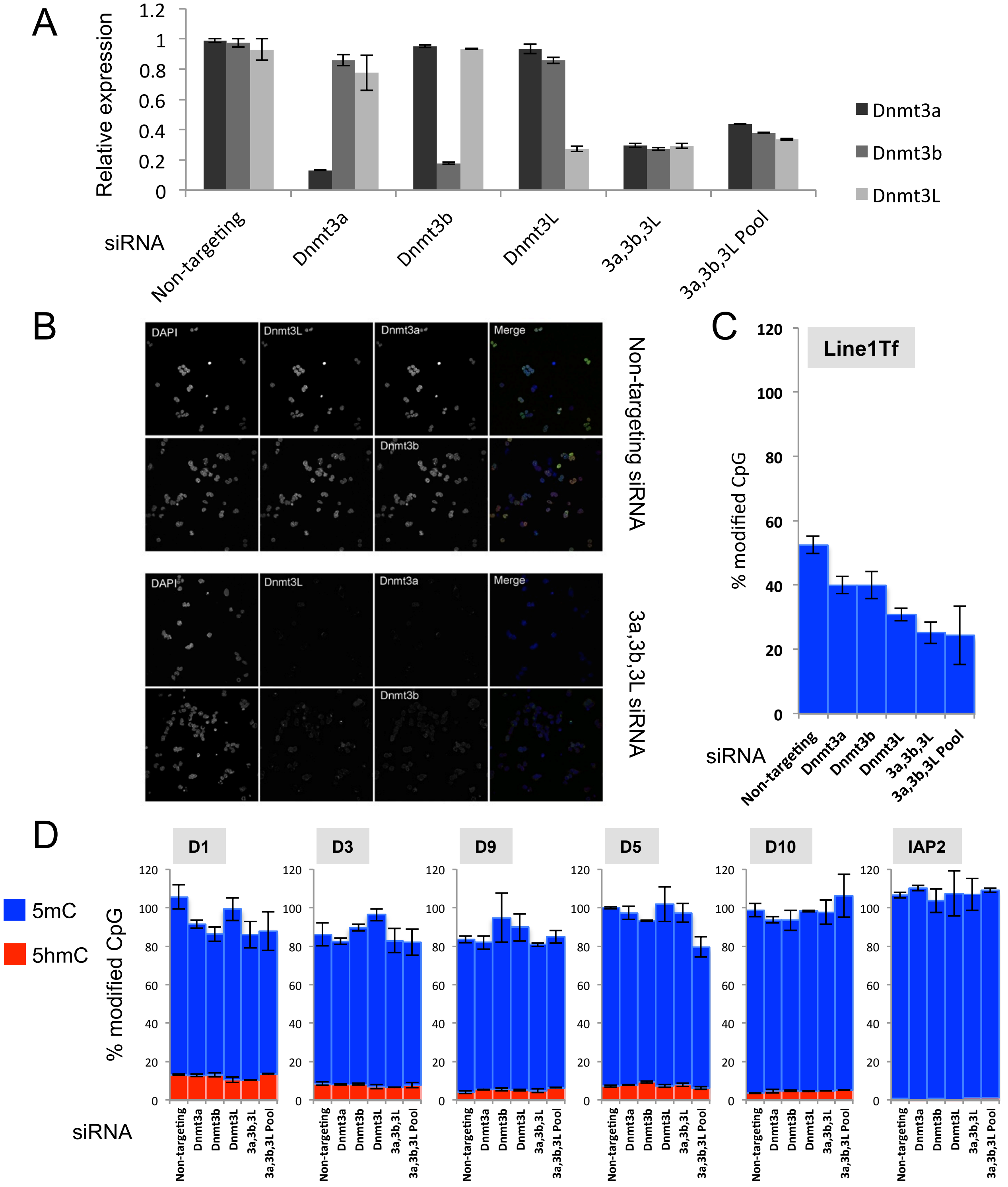
A
Relative expression
1.2
1
0.8
0.6
0.4
0.2
0
siRNA
Non-targeting
Dnmt3a
Dnmt3b
Dnmt3L
3a,3b,3L
3a,3b,3L Pool
Dnmt3a
Dnmt3b
Dnmt3L
B
DAPI
Dnmt3L
Dnmt3a
Merge
Dnmt3b
Non-targeting siRNA
3a,3b,3L siRNA
C
Line1Tf
% modified CpG
120
100
80
60
40
20
0
siRNA
D
5mC
5hmC
% modified CpG
D1
D3
D9
D5
D10
IAP2
siRNA

Figure S4. Knockdown of Dnmt3a, Dnmt3b and Dnmt3L in Serum/LIF cultured ES cells (related to Figure 3).

A. Relative expression levels of genes upon two rounds of siRNA treatment (4 days in total). Expression levels were measured by qPCR 48 hours after the second transfection. **B.** Immunofluorescence staining after siRNA downregulation of Dnmt3a, Dnmt3b and Dnmt3L. Glucosylation based MS-qPCR on Line1Tf (**C**) and on targets which demethylate in 2i (**D**) (except the last one which is resistant to demethylation in 2i). Downregulation of Dnmt3a,b and L leads to demethylation of Line1Tf sequences. In demethylating targets there is no 5hmC increase in the absence of de novo methyltransferases and a variable effect on demethylation. (Note: 3a,3b,3L Pool means SmartPool siRNA mix used for each gene). Error bars represent the range of values in two biological replicates throughout.

Table S1. Illumina sequencing run statistics
Individual biological replicates (two for RNA-seq except Day0 and three for BS-Seq) and their Illumina sequencing run statistics are displayed below. Data related to Figure 1 and Figure 4A,B.

Sample	No. of raw sequences	% Mapping efficiency	Fold coverage
**RNA-Seq**			Transcriptome length
**Day0 (RNA-Seq)**	38508073	52.4%	9.8
**Day24 Ser (1) (RNA-Seq)**	35501751	47.8%	8.2
**Day24 Ser (2) (RNA-Seq)**	36324870	42.4%	7.5
**Day24 2i (1) (RNA-Seq)**	34705282	38.9%	6.5
**Day24 2i (2) (RNA-Seq)**	26520780	35.0%	4.5
**BS-Seq**			Genome length
**Day0 (BS-Seq)**	133463876	68.8%	3.4
**Day24 Ser (1) (BS-Seq)**	139667860	71.3%	3.7
**Day24 Ser (2) (BS-Seq)**	120504243	67.0%	3.0
**Day24 Ser (3) (BS-Seq)**	138069413	67.0%	3.4
**Day24 2i (1) (BS-Seq)**	121563171	71.4%	3.2
**Day24 2i (2) (BS-Seq)**	153519229	70.2%	4.0
**Day24 2i (3) (BS-Seq)**	92612642	69.6%	2.4

Table S2. Primer list and sequences
Primers below have been used for expression analyses (*qPCR primers*) in Figure 2C, Figure 3F and Figure S4. *Gluc MS-qPCR* primers were used for CpG methylation analysis in Figure 3 and Figure S2 and Figure S4.

Primer name	Primer pair sequences (5'-3')
qPCR primers	
Dnmt3b	TGGTGATTGGTGGAAGCC<br>AATGGACGGTTGTCGCC
cMyc	CCTAGTGCTGCATGAGGAGACA<br>TCTTCTCCACAGACACCACATCA
Atp5b	GGCCAAGATGTCCTGCTGTT<br>GCTGGTAGCCTACAGCAGAAGG
Tet1	CCATTCTCACAAGGACATTCACA<br>GCAGGACGTGGAGTTGTTCA
Tet2	GCCATTCTCAGGAGTCACTGC<br>ACTTCTCGATTGTCTTCTCTATTGAGG
Dnmt3a	CCTGCAATGACCTCTCCATT<br>CAGGAGGCGGTAGAACTCAA
Dnmt3L	ATGGACAATCTGCTGCTGACTG<br>CGCATAGCATTCTGGTAGTCTCTG

Dnmt1	GGGTCTCGTTCAGAGCTG<br>GCAGGAATTCATGCAGTAAG
Uhrf1	GCTCCAGTGCCGTTAAGACC<br>CACGAGCACGGACATTCTTG
Prdm14	ACAGCCAAGCAATTTGCACTAC<br>TTACCTGGCATTTTCATTGCTC
Nanog	AAGCAGAAGATGCGGACTGT<br>ATCTGCTGGAGGCTGAGGTA
Gapdh	AACTTTGGCATTGTGGAAGG<br>ATGCAGGGATGATGTTCTGG

Gluc MS-qPCR primers	
IAP1	CTTGCTTCTTTGCACTCTGG<br>TTGTTGAAATGGAAGGGTTAGA
IAP2	AAGGTTTGAATTGGCAGAGC<br>CACTCTGGCTCCTGAAGATG
CpG-R2	AGTGAATGGCACGGACTCTA<br>CTTCCAGGCTCAGAACTGGT
CpG-D1	GCGCCTCAACTACATCTTCA<br>CGACACCACCGTTTTTATTG
CpG-D2	CGTCCACATCATAGTTCTCCTCTC<br>GAGTCAAACGCCCTCCAA
CpG-D3	CCCTAGTTGCCAAAACTGCT<br>TTCCTTCACCCTCTCTCCAC
CpG-D4	CAATCAACCTTCCTGGTGTG<br>GTGACAGGGAAACTGTGACG
CpG-D5	GAGTCCTCATCCCAGCACTT<br>AGGGAACGGGTGACTAGGT
CpG-D6	TTCCGTGTCGGGATTTCGT<br>GCGGGAGCAGGTGGATTG
CpG-D8	TGCCAGACGCTCACACAG<br>GCACGGTTTCCAATAGCAC
CpG-D9	GGCTCAGTTTCTCCAGTTCC<br>GGGTGCTGGACACATCAGTA
CpG-D10	CAGAGATGTCCCCCAATCTT<br>GCTGCAAAGGAGTGAAAGGT
CpG-D11	CCTCTAAGCCCTCGTCCTTTAGTC<br>GGAGCGCCTTGCAGACCT
CpG-Igf2r	AGAGTTCCAGGCCGCTCAAAG<br>CCTCCCTTCTCCTCTTGCTGAC
CpG-Gnas	CCCAACAAACAGCAAACATAAACA<br>GGTAGAGGCACACACACACACAAA
CpG-Line1TF	GCCTAAGCCACAGCAGCA<br>GCTGTCAGGTTCTCTGGCG

Supplementary experimental procedures

RNAi knockdown of Tet1 in Tet2KO ES cells and of Dnmt3 proteins in E14 ES cells

RNA interference experiments were performed as described (Ficz et al., 2011) with modifications. Transfections of Dharmacon siGENOME SMARTpool siRNA duplexes against mouse Tet1 (Thermo Fisher Scientific, Cat. no. M-062861-01; gcuacuaaccaagcacuua, gacgauaacuugccucaac, gaauuacaguuguuacgga, caacuugcauccacgauua) and siGENOME non-targeting siRNA#2 (Cat. no. D-001210-02; sequence not available) were done with Lipofectamine 2000 according to the manufacturer's instructions (2uL Lipofectamine, $1x10^5$ cells and 2.5uL of 20uM siRNA per well in a 12 well plate). Cells were transfected twice (with two days span between transfections) and 10 hours after the second transfection the serum/LIF medium was changed to 2i/LIF. Cells were harvested 48 hours after the 2i medium change. Transfections of Dharmacon siGENOME SMARTpool siRNA duplexes against mouse Dnmt3a (Thermo Fisher Scientific, catalogue no. M-065433-01; cgcgauuucuugagucuaa, cgaauugugucuuggugga, aaacaucgaggacauuugu, caagggacuuuaugagggu), individual siRNA duplexes against mouse Dnmt3a (Cat. no. D-065433-01; cgcgauuucuugagucuaa), SMARTpool siRNA duplexes against mouse Dnmt3b (Thermo Fisher Scientific, catalogue no. M-044164-01; gcaaugaucucucuaacgu, ggaaugcgcuggguacagu, uaaucuggcuaccuucaau, gcaaagguuuauaugaggg), individual siRNA duplexes against mouse Dnmt3b (Cat. no. D-044164-01; gcaaugaucucucuaacgu), SMARTpool siRNA duplexes against mouse Dnmt3L (Thermo Fisher Scientific, catalogue no. M-063056-01; gaagacaucugccucugcu, gguacgaagucaaagugaa, cgacagcucuagcccugau, cgacaggcagagagaugau) and siGENOME non-targeting siRNA#2 (catalogue no. D-001210-02; sequence not available) were done with Lipofectamine 2000 according to the manufacturer's instructions (3uL Lipofectamine, $1x10^5$ cells and 2.5uL of 20uM siRNA per well in a 12 well plate) in Serum/LIF cultured ES cells for two rounds. DNA was isolated from cells 48h after the second siRNA transfection and analysed.

Immunofluorescence, microscopy and image analysis

Antibody staining of DNA methylation (Eurogentec, BI-MECY) and hydroxymethylation (Active Motif, 39769) was performed as previously described (Santos et al., 2003) with modifications. Briefly, cells were fixed with 4% PFA for 15 minutes and, after permeabilisation with 0.5% Triton X-100, the samples were treated with 4N HCl for 10 minutes at room temperature, washed in PBS/Tween and blocked overnight; simultaneous incubation with both primary antibodies followed by simultaneous secondary detection was used. For DNMT3a (Abcam ab13888), DNMT3b (Abcam ab 13604) and DNMT3L (kind gift from Shoji Tajima) staining, same procedure was followed but no HCl treatment was performed. Mouse blastocysts were fixed and permeabilised as before and stained for NANOG (Abcam, ab21603), DNMT3B (Abcam, ab13604) and TET1 (C-terminus; a kind gift from Kristian Helin). Single optical sections were captured with a Zeiss LSM510 Meta microscope (63x oil-immersion objective) and the images pseudo-coloured using Adobe Photoshop. RGB profiles were plotted with ImageJ 1.44p (NIH) and fluorescence semi-quantification analysis performed with Volocity 5.5 (Improvision).

Mass spectrometry of nucleosides

Genomic DNA was digested using DNA Degradase Plus (Zymo Research) according to the manufacturer's instructions and analyzed by liquid chromatography-tandem mass spectrometry on a LTQ Orbitrap Velos mass spectrometer (Thermo Scientific, Hemel Hempstead, UK) fitted with a nanoelectrospray ion-source (Proxeon, Odense, Denmark). Mass spectral data for C, 5mC and 5hmC were acquired in high resolution full scan mode (R >40,000 for the protonated pseudomolecular ions and >50,000 for the accompanying protonated base fragment ions), and also in selected reaction monitoring (SRM) mode. SRM data, monitoring the transitions 228 → 112.0505 (C), 242 → 126.0662 (5mC) and 258 → 142.0611 (5hmC), were generated by HCD fragmentation using a 10 mass unit parent ion isolation window, a relative collision energy of 20% and R >14,000 for the fragment ions. Peak areas for the fragment ions were obtained from extracted ion chromatograms of the relevant scans and quantified by external calibration relative to standards obtained by digestion of nucleotide triphosphates.

Luciferase reporter assays

An 8.7 kb fragment covering the Dnmt3b promoter and upstream region (p3b -8615/+93) (Ishida et al., 2003) was a kind gift from Kiyoe Ura. p3b -8615/+93Δ was generated by removing a 2kb fragment from p3b -8615/+93 by SpeI digestion. The 1 kb fragment surrounding the Dnmt3b promoter was cloned into pGL3-basic (Promega). For transient transfection assays, ESCs were cultured in either serum or 2i based complete media for at least 3 passages and $1x10^5$ cells were co-transfected with 0.8 µg pGL3 firefly luciferase vector and 0.08 µg pRL-TK renilla luciferase vector in 24-well plates in 2-4 replicates using Fugene6 (Promega). After 40 hours, firefly and renilla luciferase activities were measured in the cell lysate using the Promega dual-luciferase reporter assay system and a Microlumat Plus LB96V luminometer.

Western blot analysis

Whole cell protein extracts were isolated using 1xRIPA buffer (Thermo Scientific, 89900) with protease and phosphatase inhibitors (Fisher Scientific, PN87786 and PN78420). 10 µg of proteins were resolved by SDS-PAGE and transferred on nitrocellulose membranes. Membranes were blocked overnight in PBS-0.1%Tween (PBST) containing 5% BSA (blocking buffer). Primary antibody incubation was done at room temperature for 2 hours (Anti-DNMT3B: Abcam ab13604; Anti-DNMT3A: Abcam ab13888; Anti-UHRF1: Santa Cruz Biotech. sc-98817). Membranes were washed in PBST and incubated with HRP conjugated secondary antibodies in blocking buffer. HRP conjugates were detected with enhanced chemiluminescence (ECL, Amersham Biosciences).

BS-Seq Analysis

Raw sequence reads were trimmed to remove both poor quality calls and adapters using Trim Galore (www.bioinformatics.babraham.ac.uk/projects/trim_galore/) (v0.2.2, default parameters). Remaining sequences were mapped to the mouse NCBIM37 genome using Bismark (Krueger and Andrews, 2011) (v0.7.4, default parameters), and CpG methylation calls were extracted and analysed using SeqMonk (www.bioinformatics.babraham.ac.uk/projects/seqmonk/) and custom R scripts. Methylation over a given genomic feature was calculated by averaging the individual methylation levels of CpGs covered by at least 3 reads and only features with at least 3 CpGs were used. For comparison with RRBS data (Smith et al., 2012; Seisenberger

et al., 2012), CpG islands with at least 3 CpGs covered by at least 5 reads in the RRBS datasets were selected and further filtered to exclude CpG islands covered by fewer than 100 reads in all BS-seq and RRBS datasets. CpG island annotations were used based on pull down experiments (Illingworth et al., 2010). Promoters were defined as the region -1 kb to +500 bp of the transcription start site as annotated in NCBIM37. ICR coordinates were used from E12.5 embryos (Tomizawa et al., 2011). Repeat annotations were extracted from the UCSC RepeatMasker track (mm9 build). For major satellite methylation analysis Bismark was used to map all reads against the mouse major gamma satellite consensus sequence and the methylation calls from these results were analysed directly. Hierarchical clustering of CpG island methylation patterns was performed using "heatmap.2" from the gplots package in the R statistics program. The distance matrix was calculated based on Pearson's correlation across all samples and the cluster generating method was complete. To assess the relative support for the node of each cluster, bootstrap resampling was performed 10,000 times using the pvclust package (Suzuki and Shimodaira, 2006).

RNA-Seq Analysis

RNA-Seq data was mapped to the mouse NCBIM37 genome assembly using TopHat (v1.4.1, options -g 1) in conjunction with gene models from Ensembl release 61. Initial quantitation was made by counting the number of reads per transcript corrected per million reads (RPM). This was adjusted by globally matching the count distributions at the 75th percentile. Expression in Fig. 2B was calculated using the RPKM pipeline of the Seqmonk software.

Oxidative Hairpin bisulfite analysis

Hairpin bisulfite analysis was performed as previously described with slight modifications(Arand et al., 2012). 1 µg of DNA was digested with BsaWI (for L1) or DdeI (for IAP) for 3h and subsequently ligated with the hairpin linker (Arand et al., 2012). The ligation mix was washed 3x with 500 µl ddH2O on YM-30 Microcon columns (Millipore) and eluted in 30 µl ddH2O. 20 µl were oxidized using a prototype hydroxymethylation detection kit obtained by courtesy of Cambridge Epigenetix (www.cegx.co.uk, see also Booth et al., 2012) and the remaining DNA stored for bisulfite treatment. Bisulfite treatment of the oxidized and non-oxidized sample was performed as described in (Arand et al., 2012). Proper oxidation was validated by amplification of a hydroxymethylated spike-in oligo added to each sample before oxidation and further restriction analysis according to Cambridge Epigenetix. L1 and IAP elements were amplified with HotFire Polymerase (SolisBioDyne) with following specific primers attached to the TruSeq adaptor sequence (Illumina) and PCR conditions: IAP F: TTTTTTTTTTAGGAGAGTTATATTT, R: ATCACTCCCTAATTAACTACAAC, 43 cycles 95°C 1 min, 51 °C 1.30 min, 72°C 1 min, L1 F: TGGTAGTTTTTAGGTGGTATAGAT, R: TCAAACACTATATTACTTTAACAATTCCCA,
45 cycles 95°C 1 min, 55°C 45s, 72°C 1.30 min. The PCR products were gel purified, amplified with TruSeq adaptor sequences with single read indexes (5 cycles) and sequenced on a Illumina MiSeq. The data were processed using BiQAnalyzerHT (ref (Lutsik et al., 2011)) and python scripts.

References

Booth, M. J., Branco, M. R., Ficz, G., Oxley, D., Krueger, F., Reik, W., and Balasubramanian, S. (2012). Quantitative Sequencing of 5-Methylcytosine and 5-Hydroxymethylcytosine at Single-Base Resolution. Science *336*, 934–937.

Illingworth, R. S., Gruenewald-Schneider, U., Webb, S., Kerr, A. R. W., James, K. D., Turner, D. J., Smith, C., Harrison, D. J., Andrews, R., and Bird, A. P. (2010). Orphan CpG Islands Identify Numerous Conserved Promoters in the Mammalian Genome. PLoS Genet *6*, e1001134.

Ishida, C., Ura, K., Hirao, A., Sasaki, H., Toyoda, A., Sakaki, Y., Niwa, H., Li, E., and Kaneda, Y. (2003). Genomic organization and promoter analysis of the Dnmt3b gene. Gene *310*, 151–159.

Krueger, F., and Andrews, S. R. (2011). Bismark: a flexible aligner and methylation caller for Bisulfite-Seq applications. Bioinformatics *27*, 1571–1572.

Lutsik, P., Feuerbach, L., Arand, J., Lengauer, T., Walter, J., and Bock, C. (2011). BiQ Analyzer HT: locus-specific analysis of DNA methylation by high-throughput bisulfite sequencing. Nucleic Acids Research *39*, W551–W556.

Santos, F., Zakhartchenko, V., Stojkovic, M., Peters, A., Jenuwein, T., Wolf, E., Reik, W., and Dean, W. (2003). Epigenetic Marking Correlates with Developmental Potential in Cloned Bovine Preimplantation Embryos. Current Biology *13*, 1116–1121.

Suzuki, R., and Shimodaira, H. (2006). Pvclust: an R package for assessing the uncertainty in hierarchical clustering. Bioinformatics *22*, 1540–1542.
